# Sources of variability in auditory brainstem response thresholds in a mouse model of noise-induced hearing loss

**DOI:** 10.1121/10.0016593

**Published:** 2022-12-15

**Authors:** Katrina M. Schrode, Micheal L. Dent, Amanda M. Lauer

**Affiliations:** 1 Department of Otolaryngology–Head and Neck Surgery and Center for Hearing and Balance, Johns Hopkins University School of Medicine, 515 Traylor Building, 720 Rutland Avenue, Baltimore, Maryland 21205, USA; 2 Department of Psychology, B76 Park Hall, University at Buffalo-SUNY, Buffalo, New York 14260, USA

## Abstract

Numerous and non-acoustic experimental factors can potentially influence experimental outcomes in animal models when measuring the effects of noise exposures. Subject-related factors, including species, strain, age, sex, body weight, and post-exposure measurement timepoints, influence the observed hearing deficits. Experimenter effects, such as experience with experimental techniques and animal handling, may also factor into reported thresholds. In this study, the influence of subject sex, body mass, age at noise exposure, and timepoint of post-exposure recording are reported from a large sample of CBA/CaJ mice. Auditory brainstem response (ABR) thresholds differed between noise-exposed and unexposed mice, although the differences varied across tone frequencies. Thresholds across age at noise exposures and measurement delays after exposure also differed for some timepoints. Higher body mass correlated with higher ABR thresholds for unexposed male and female mice, but not for noise-exposed mice. Together, these factors may contribute to differences in phenotypic outcomes observed across studies or even within a single laboratory.

## I. INTRODUCTION

Laboratory mice (*Mus musculus*) are common animal models in the field of communicative disorders and hearing primarily because they have relatively inexpensive husbandry requirements and are amenable to genetic manipulations. The effects of age and noise are common experimental variables in these types of studies, which often correlate measures of hearing with pathologies of the peripheral and central auditory system (e.g., Kobrina *et al.*, 2020; Schrode *et al.*, 2018). The auditory brainstem response (ABR) is one of the quickest ways to measure thresholds for a broad range of frequencies in a relatively short time period. An ABR audiogram can be conducted on an anesthetized mouse in about 30–45 min. Furthermore, the same animal can be measured at multiple timepoints in their life, minimizing inter-subject variability to instead allow a focus on key factors of interest, such as the effects of harmful noise or the aging process. ABRs can be reliably performed on mice young and old, a claim that is difficult to make for the sometimes-preferred behavioral methodologies due to long training periods (e.g., Kim *et al.*, 2022).

Mouse ABRs have been measured since the late 1970s (Henry, 1979) and have provided a wealth of information about the genetics and physiology of auditory function and dysfunction. Laboratory mouse ABR audiograms are known to differ by as much as 80 dB at the same frequencies across strains [reviewed in Dent *et al.* (2018)]. There are also known developmental differences in ABR measurements, with thresholds in one laboratory mouse strain improving by about 70 dB between the 13th and 18th day of a mouse's life (Song *et al.*, 2006). Thresholds also significantly decline as mice age, and the rate of decline differs across strains and sexes [reviewed in Screven and Dent (2021)]. Several recent reports have highlighted the need for consistency, training, and experimental transparency when conducting rodent ABRs to minimize errors and to maximize replicability (Domarecka *et al.*, 2021; Kim *et al.*, 2022).

When ABR measurements in the same strain of mouse differ across studies, it is often difficult to understand the source of the differences. A recent study highlighted a number of variables known to influence rat ABRs (Domarecka *et al.*, 2021), and there is no reason to doubt that they influence mouse ABRs as well. Unfortunately, the variables that may be affecting the results are often not reported in publications. Measurements can be taken from mice of different ages, sexes, and sizes, to name just a few variables. Other factors that may contribute to differences across studies, such as husbandry conditions and the ambient acoustics in the housing environment, are typically not considered in the context of noise exposure outcomes in mice. Our previous work has shown that acoustic conditions in the housing rooms can affect the auditory phenotype of mice (Lauer and May, 2011; Wu *et al.*, 2020). These same factors can affect ABR measurements following noise exposures. Noise exposures can be conducted at different timepoints in a mouse's life, and then post-exposure ABRs can be taken immediately or following short or long delays. Differences in the noise exposure conditions a mouse experiences may skew threshold shifts in unknown ways.

Further, the ABR procedure relies on accurate and consistent placement of needle electrodes and the determination of waveform peaks by experimenters. Experimenters in a single study may differ widely in their experience with the procedures, which may lead to differences in the reported thresholds in that study. Kim *et al.* (2022) outline best practices for and variables that can affect ABR data collection, which include speaker calibration, animal body temperature, anesthesia level, and system noise. Thus, it is not simply animal factors that can affect mouse ABRs, but researcher and equipment factors as well. Researchers not careful in their approach to conducting ABRs may significantly affect reported thresholds.

In the present study, we leveraged our laboratory's large ABR dataset to further elucidate extraneous factors that affect ABR measurements and noise exposure outcomes in mice. First, we compared ABR thresholds in exposed and unexposed male and female CBA/CaJ mice. We next compared thresholds for mice exposed at two different age groups and three different post-exposure measurement timepoints. We also compared baseline hearing and noise-induced hearing loss as a function of sex and weight. Next, we sought to establish whether baseline hearing measurements were similar across both highly experienced and less experienced experimenters. Finally, we compare ABR audiograms from those four experimenters. We found that some subject and experimenter variables led to differences in measures of hearing at baseline and following a noise exposure, while other variables were less influential.

## II. MATERIALS AND METHODS

### A. Ethics statement

All procedures were approved by the Johns Hopkins School of Medicine Institutional Animal Care and Use Committee (IACUC) and were in accordance with the *Guide for Care and Use of Laboratory Animals.*

### B. Subjects

Our archival dataset includes ABR measurements performed by different experimenters in unexposed and noise-exposed adult male and female CBA/CaJ mice using the same experimental apparatus, stimulation, and recording parameters. This strain is commonly used as the “normal-hearing” strain in auditory research. Subjects were obtained from Jackson Laboratory (stock #000654) or bred in our colony. A total of 171 mice were used across experiments. Subject numbers in specific experimental conditions are noted in the figure legends. Mice ranged in age from 1 to 20 months. Thresholds from these animals are compared to data we previously reported for aged, unexposed mice that were obtained by one of our experienced experimenters using identical procedures and equipment (Kobrina *et al.*, 2020).

Animals were group-housed up to five per cage in static filter-top shoebox cages filled with corncob bedding and kept in a quiet, low-traffic room to minimize extraneous noise exposure (Lauer *et al.*, 2009; Wu *et al.*, 2020). On occasion, male mice were separated from cagemates at the recommendation of a veterinarian due to fighting behavior. Room lights were kept on a 12/12 h day/night cycle (lights on 07:00–19:00), and a window in the room provided natural light. Animals were given *ad libitum* access to food and water and provided with nesting material and small tubes for enrichment. Animal housing room temperature and humidity were maintained at 18 °C–26 °C and 30%–50%, respectively. Animals were transported in their home cages to the experiment rooms at least 15 min prior to testing. Experiment rooms were kept at approximately the same temperature and humidity levels as the housing room and were located within the same laboratory suite.

Animals were randomly assigned to test and exposure conditions. Since aging mice are larger and show obviously altered body conditions, it was not possible to blind the experimenters to age at exposure or testing. Similarly, it quickly becomes obvious to an experimenter if a mouse is noise-exposed due to the reduced response amplitudes and increased thresholds, so blinding to exposure condition was not feasible. Reliable otoscopic examination of the tympanic membranes to screen for external or middle ear disease was not possible, but post-experiment tissue harvest has revealed evidence of ear infections, obstructions, or perforations in CBA/CaJ mice fewer than five times over the course of 15 years in our laboratory. Thus, we assume that external or middle ear obstructions were not present in the animals used in the current experiment.

### C. Noise exposure

Noise exposures and sham exposures were performed either at age 1–2 months or 3–7 months. Procedures were similar to those described by Schrode *et al.* (2018). For both noise and sham exposure conditions, awake mice were placed individually in a small wire cage mounted inside a small sound-attenuating booth [Industrial Acoustic Company Inc. (IAC), Bronx, NY] below two speakers (TW57; Pyramid Audio, Brooklyn, NY). Mice selected at random were exposed to broadband noise (peak energy at 2–50 kHz) generated using custom matlab-based software or a sham condition (no noise broadcast) for 2 h while rotating gradually through the sound field. We calibrated the noise to 100 dB sound pressure level (SPL) at the location of the cage prior to exposure using a 1/2-in. free-field microphone and Z-weighting (SoundTrack LxT; Larson Davis, DePew, NY). Although a recent study showed no day/night effects on noise exposure outcomes in the CBA/CaJ strain (Versteegh *et al.*, 2022), all exposures were performed during the normal work hours of 08:30–17:30, Monday through Friday.

### D. Experimenters

Experimenters 1–4 were all highly experienced in the ABR measurement technique. The “Other” group included ABRs measured by four inexperienced experimenters, such as summer students and new technicians, each only testing a small number of mice. Experimenter 2 performed many of the noise exposures and recordings over the course of several years, whereas the Other group performed very few noise exposures and ABR recordings.

### E. ABR

We recorded ABRs at several time points in unexposed mice or following noise exposure (1, 2–5, and 19–20 months) and at 1 month following sham exposure. Procedures were similar to previous reports (McGuire *et al.*, 2015; Lauer, 2017; Schrode *et al.*, 2018; Kobrina *et al.*, 2020; Kim *et al.*, 2022). Briefly, mice were weighed and anesthetized with 100 mg/kg ketamine and 10 mg/kg xylazine (intraperitoneal), and veterinary ophthalmic ointment was applied to the eyes to prevent corneal abrasions. Animals were placed inside a small sound-attenuating chamber (IAC) with a built-in Faraday cage and lined with SONEX acoustic foam (pinta acoustic, Inc., Minneapolis, MN) to reduce acoustic reflections. Animals were maintained at 37 ± 1 °C using a custom feedback-controlled heating pad and rectal temperature probe. Mice were placed at 0° facing a free-field speaker (FT28D; Fostex, Tokyo, Japan) positioned 30 cm from the vertex of the skull. Low-impedance subcutaneous platinum needle electrodes disinfected with 70% ethanol (Grass, obtained from Natus, Middleton, WI) were inserted over the left mastoid and at the vertex of the skull, and a ground electrode was inserted into the leg muscle. Animals were monitored for increased or slowed heart rate and breathing, and supplemental anesthesia doses of 1/3 to 1/2 the original dose were given if signs of whisking or other movement were detected.

Stimulus generation, presentation, and response acquisition were controlled using custom matlab-based software, a Tucker-Davis Technologies (Alachua, FL) RX6 multifunction processor, and a personal computer (PC). We used custom matlab software to calibrate stimuli using a 1/4-in. free-field microphone (type 4939; Brüel and Kjær, Nærum, Denmark) placed at the location of the mouse's head. Responses were averaged over 300 repetitions, sampled at 9.5 kHz, and bandpass filtered from 300 to 3000 kHz. Stimuli consisted of clicks (0.1 ms square wave pulse of alternating polarity) and 5-ms tones at frequencies of 6, 8, 12, 16, 24, and 32 kHz (0.5 ms cos^2^ onset/offset), generated with a sampling frequency of 195 kHz, and presented at a rate of 20/s. Clicks were tested principally to verify the presence of a response.

We presented a given stimulus frequency at descending levels starting at 85–105 dB (depending on frequency and the output capabilities of the speaker) until the response could not be discerned from the baseline physiological noise. An automated statistical threshold estimation procedure implemented in a custom software was used to reduce potential bias in determining ABR thresholds [interpolated at 2 standard deviations (SD) above baseline noise]. The accuracy of this method and overall response recording quality were spot-checked by an experienced observer. Threshold was defined as the sound level at which the ABR peak-to-peak (any peak) amplitude was 2 SD above the average baseline noise amplitude during the period of the recording when no sound stimulus was present. This method provides an unbiased and consistent threshold estimate across experimenters. Threshold data are available upon request, subject to compliance with institutional data-sharing policies. Once one tone was presented at all stimulus intensities, the next tone was presented, and so on, in a pseudorandom order. Testing lasted approximately 40–60 min per session. Mice were returned to their home cages following testing and monitored until they were able to reach food and water prior to being returned to the animal housing room. Some animals were tested at multiple timepoints pre- and post-exposure, typically with at least 1 month between test sessions.

### F. Statistical analysis

Data were analyzed in R using the *lme4* and *afex* packages (Bates *et al.*, 2014; Singmann *et al.*, 2020). A general linear mixed model was used to test for differences in threshold considering age of the animal at the time of noise exposure and the delay until ABR testing. We included a “group” variable with the categories (1) exposed at 1–2 months old and tested 1 month later; (2) exposed at 1–2 months and tested 2–5 months later; (3) exposed at 1–2 months and tested 19–20 months later; and (4) exposed at 3–7 months old and tested 1 month later. We also included frequency in the model, along with the interaction between group and frequency. Sex was not included in this model, as we did not test enough individuals of each sex in each age group to draw meaningful conclusions. *Post hoc* Tukey tests were used to determine which group means differed.

We also tested whether there was a significant correlation between threshold and subject mass (weight), since we have anecdotally observed that smaller animals tend to have more robust ABRs with better signal-to-noise ratios. For this analysis, we calculated the pure tone average for each individual based on thresholds in response to tones of 8, 16, and 32 kHz. We also limited the analysis to young animals and, among exposed subjects, focused on the group exposed at 1–2 months and tested 1 month later to avoid confounds introduced by different exposure and testing schedules.

Another general linear mixed model was used to test for statistical differences in thresholds when comparing experimenters, subject sex, and noise exposure status in animals less than 90 days of age. The model included stimulus frequency, experimenter, subject sex, and exposure status as categorical main effects and subject ID as a random effect to account for repeated measurements in the same animal. We also included all two-way interactions except the interaction between sex and experimenter, which we had no theoretical reason to include. We followed up significant interactions with *post hoc* Tukey tests. A *p* value of 0.05 was used to determine significance for all analyses.

## III. RESULTS

**FIG. 1.**
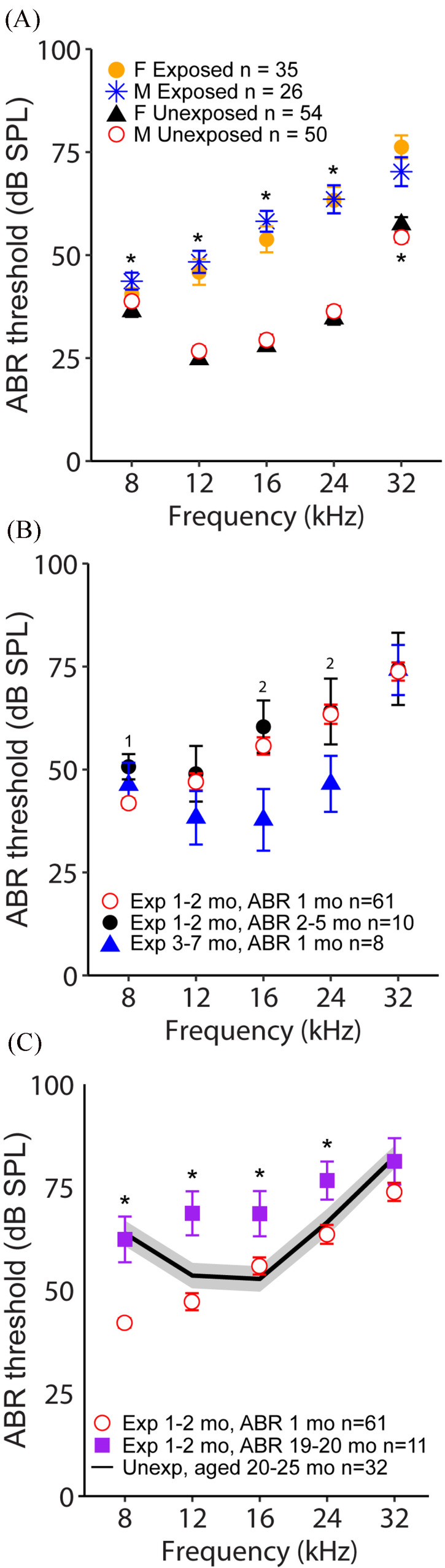
(Color online) (A) ABR thresholds at 1 month after exposure or sham exposure for young adult male and female mice. Symbols denote means, and error bars denote SEMs. Asterisks indicate significant differences between males and females. (B) ABR thresholds measured in mice of different ages at exposure and time of post-exposure ABR testing. Symbols denote means, and error bars denote SEMs (note that some error bars are smaller than the symbols). Numbers indicate a significant difference between the following groups: 1, experiments (Exp) 1–2 months (mo), ABR 1 month vs experiments 1–2 months, ABR 2–5 months; 2, experiments 1–2 months, ABR 2–5 months vs experiments 3–7 months, ABR 1 month. (C) ABR thresholds measured in young and old mice exposed at the same age and unexposed mice. Data from old, unexposed mice shown in (C) are replotted from our previous work (Kobrina *et al.*, 2020). Symbols denote means, and error bars denote SEMs. For some data points, the small error bars are obscured by the symbols. Asterisks denote significant differences between exposed groups.

Mice exposed to noise at 1–2 months of age had higher thresholds overall compared to unexposed animals at 1 month post-exposure, as expected [Fig. 1(A)]. In the statistical model, there were significant interactions between exposure status and frequency [*F*(4556 = 87.09, *p* < 0.0001], sex and frequency [*F*(4,555) = 3.84, *p* = 0.0043], and exposure status and sex [*F*(1,475) = 13.36, *p* = 0.0003]. Noise-exposed males had significantly higher thresholds than noise-exposed females at all frequencies except 32 kHz (all *t* ≥ 2.82, all *p* ≤ 0.0256). There were no statistically significant sex differences in unexposed animals except for 32 kHz [Fig. 1(A)].

When comparing across ages, exposure timepoints, and post-exposure recording timepoints, the age at exposure yielded greater differences in ABR thresholds than the time after exposure in young adult mice [Fig. 1(b)], and the interaction between age/exposure group and frequency was significant [*F*(12 318) = 3.09, *p* = 0.0004], indicating that differences between groups were dependent on frequency (Table I). Mice exposed at 3–7 months of age and tested 1 month later showed better thresholds than mice exposed at 1–2 months of age and tested either 1 month (same duration after noise exposure) or 2–5 months (same age range) later. However, these differences were only statistically significant between those exposed at 3–7 months and those tested 2–5 months after exposure at 1–2 months of age, and only at 16 and 24 kHz. Mice exposed at 3–7 months were, thus, more resistant to noise exposure in the mid-frequency range than those exposed at younger ages.

**TABLE I.** Analysis of variance (ANOVA) results of linear mixed model comparing ABR thresholds by age of exposure and ABR test.

Effect	NumDF	DenDF	*F* value	*p* value	Partial *ε*^2^
Frequency	4	318	44.6	<0.0001	0.35
Exposure age group	4	160	22.3	<0.0001	0.17
Frequency: exposure age group	12	318	3.1	0.0004	0.10

In contrast, mice exposed at 1 month of age and tested in old age (19–20 months after exposure) showed substantially elevated thresholds compared to the young adult exposed mice [Fig. 1(C)]. This young-exposed, tested in old age group had significantly higher thresholds than the group noise-exposed at 3–7 months and tested 1 month later at frequencies 12–24 kHz (all *t* ≥ 4.15, all *p* ≤ 0.0003) and significantly higher thresholds than the group noise-exposed at 1 month and tested 2–5 months later at all frequencies <32 kHz (all *t* ≥ 4.2, all *p* ≤ 0.0002). This effect could not be attributed solely to age, as unexposed, aged mice showed lower thresholds for mid-frequencies compared to aged, exposed mice [Fig. 1(C); unexposed data from Kobrina *et al.* (2020)].

**FIG. 2.**
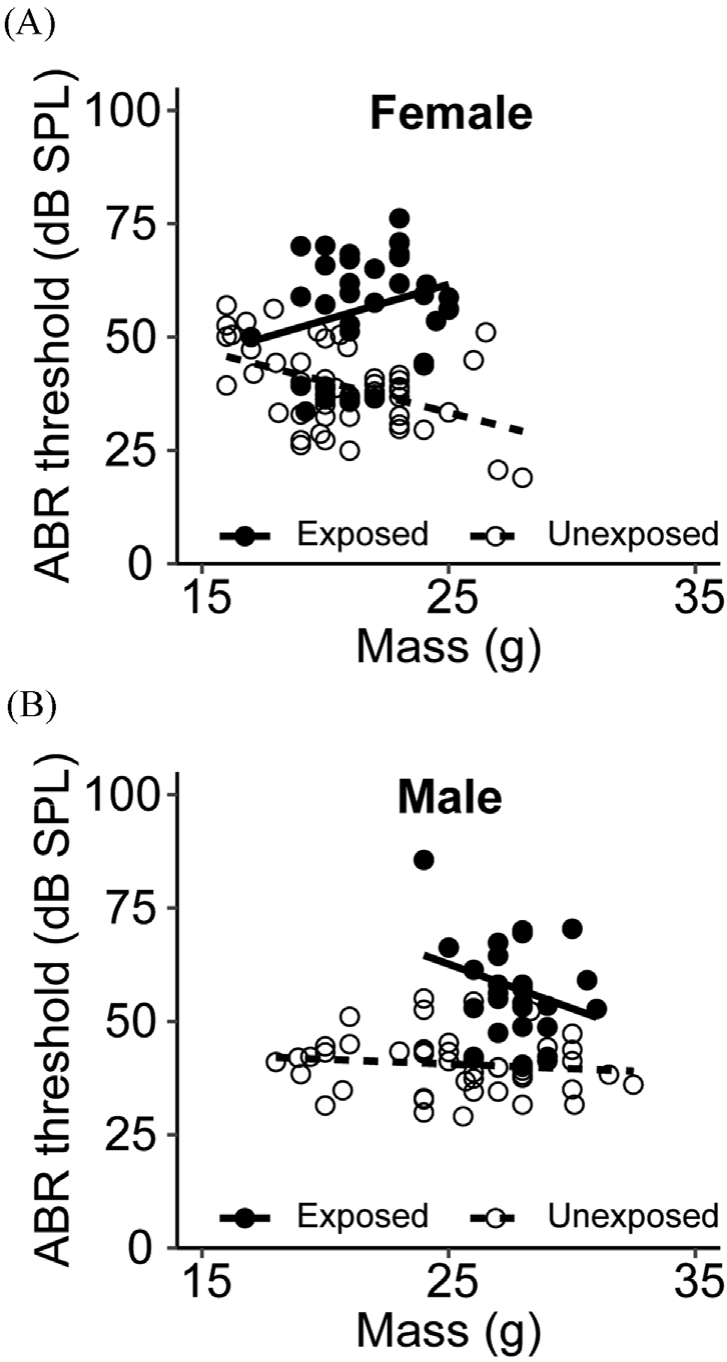
Relationship between pure tone average threshold and body mass for females (A) and males (B). Symbols denote individual mice. Regression lines indicate best fit. Open symbols and dashed line, unexposed; closed symbols and solid line, exposed.

Among young mice, heavier unexposed females showed lower pure tone averages than lighter animals. There was a significant negative correlation between body mass and pure tone average threshold for unexposed females, but not males (female: *ρ* = −0.27, *p* = 0.0401; male: *ρ* = −0.12, *p* = 0.4027; Fig. 2). Across all subjects, noise-exposed animals had higher thresholds compared to unexposed animals of similar mass. Among noise-exposed animals, females showed a non-significant weakly positive correlation, while males showed a non-significant weakly negative correlation (female: *ρ* = 0.28, *p* = 0.0992; male: *ρ* = −0.30, *p* = 0.1301). Correction for the minor variation in age in these animals (ranging from 1 to 3 months) did not qualitatively change the results.

**FIG. 3.**
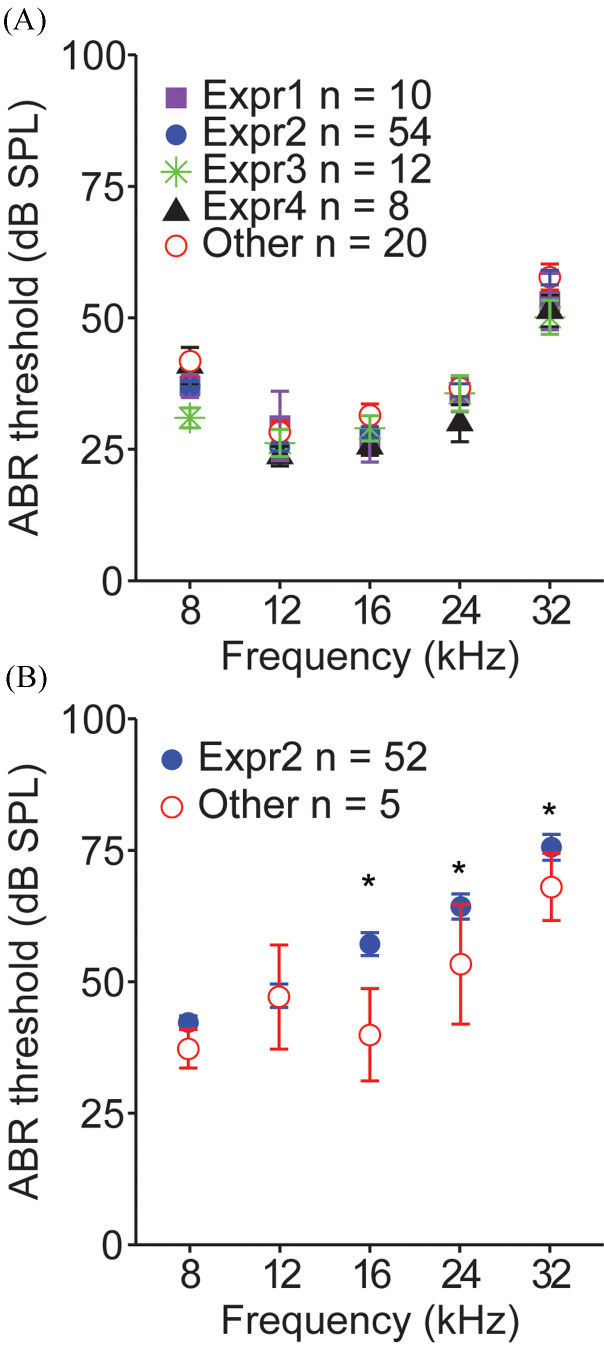
(Color online) (A) Comparisons of ABR thresholds by experimenter (Expr). There were no differences between experimenters for thresholds measured in 1–2-month-old young adult, unexposed mice. Symbols denote means, and error bars denote SEMs. (B) ABR thresholds measured in 1–2-month-old young adult mice 1 month after noise exposure. Symbols denote means, and error bars denote SEMs. Asterisks indicate significant differences between experimenters.

There were no statistically significant differences in ABR thresholds measured in young (1–2-month-old) unexposed animals when comparing across experimenters [Fig. 3(A)]. Variability of thresholds for each experimenter, expressed as standard error of the mean (SEM) bars on the figures, was also low. In contrast, thresholds measured in young noise-exposed animals (exposed at 1–2 months, tested 1 month later) did differ by experimenter for frequencies of 16 kHz and above [Fig. 3(B)], resulting in significant interactions between experimenter and noise exposure status [Table II; *F*(2,392) = 11.34, *p* < 0.0001] and between experimenter and frequency [Table II; *F*(16 557) = 1.68, *p* = 0.0468].

**TABLE II.** ANOVA results of linear mixed model comparing ABR thresholds in CBA/CaJ mice.

Effect	NumDF	DenDF	*F* value	*p* value	Partial *ε*^2^
Frequency	4	556	87.09	<0.0001	0.37
Sex	1	136	2.52	0.1146	0.01
Exposure status	1	375	61.52	<0.0001	0.09
Experimenter	4	204	1.40	0.2355	0.01
Frequency:sex	4	555	3.84	0.0043	0.03
Frequency:exposure status	4	553	38.69	<0.0001	0.21
Frequency:experimenter	16	557	1.68	0.0468	0.04
Sex:status	1	475	13.36	0.0003	0.02
Status:experimenter	2	392	11.34	<0.0001	0.04

## IV. DISCUSSION

A recent focus on various factors affecting the outcomes of ABR experiments reporting threshold changes after noise exposures led to this report. We found that noise-exposed mice had higher thresholds than non-exposed mice a month after exposure. While there were sex differences in the noise-exposed mice, with males having higher thresholds than females, there were no sex differences between unexposed males and females. Our finding that male mice showed significantly higher post-exposure thresholds than females suggests differential effects of noise exposures that are missed when only one sex of mice is used. Female and male mice showed similar variability in ABR thresholds whether they were unexposed or exposed. Many previous noise exposure studies have used only male animals under the belief that females will show more variability due to the estrous cycle; however, our data show that variability expressed as SEMs was not substantially greater in one sex vs the other for unexposed or noise-exposed mice.

Another significant finding was that mice exposed later in adulthood (3–7 months) had less hearing loss after noise exposure than mice exposed earlier in adulthood (1–2 months), consistent with previous reports (Ohlemiller *et al.*, 2000; Ohlemiller *et al.*, 2011). Furthermore, mice subjected to noise exposure at early ages showed large deficits when recorded at 19–20 months compared to earlier recording timepoints and age-matched unexposed mice, confirming that age and noise can have additive effects on hearing loss. These results also suggest that recordings should be taken at multiple timepoints to fully understand the damaging effects of noise, as observed in mouse models of noise-induced cochlear synaptopathy (Kujawa and Liberman, 2006; Fetoni *et al.*, 2022).

Larger body mass was associated with decreased thresholds in unexposed female mice. The trend toward increased sensitivity may be a result of continued development of the auditory system in these young animals. However, larger body mass was not associated with increased thresholds in noise-exposed animals, possibly due to abnormal weight ranges in the noise-exposed mice that likely experienced stress interactions.

We also demonstrated that the experimenter may influence susceptibility to noise exposure as assessed via ABRs. When ABR audiograms were measured for young, unexposed mice by experienced experimenters along with a group of inexperienced researchers, they did not significantly differ at any frequency [Fig. 3(A)]. This suggests that the statistical threshold calculation procedures used by our laboratory are accurate and robust. However, when considering noise-exposed mice, differences emerged between ABR thresholds measured by experimenter 2, who exposed and recorded from the greatest number of noise-exposed animals, and the inexperienced researchers, at least at the three highest frequencies tested. The reasons for this are not entirely known; however, we suspect that less experienced experimenters cause more stress to the animals when they are handled, and this is protective against noise damage. Exposure to restraint stress prior to noise exposure has previously been shown to protect against cochlear damage in mice (Meltser *et al.*, 2009).

As a whole, the results here suggest that adequately trained experimenters can produce reliable ABR threshold measurements in mice, but that factors such as sex, age at noise exposure, post-exposure recording timepoint, and experimenter experience play a large role in noise exposure outcomes. We did not compare ABR audiograms for other potentially important variables, such as husbandry conditions, equipment, test-retest reliability differences across experimenters, time of testing, and experimental designs, but all of these variables need to be considered when attempting to interpret outcomes of noise exposures in mice across studies. In addition to ensuring that experimenters are adequately trained and practiced and that experimental conditions are thoroughly documented, studies should also consider the potential effects of the experimenter's sex on hearing outcomes in mice (Sorge *et al.*, 2014; Georgiou *et al.*, 2022). Future studies are needed to systematically address the influence of these variables on ABRs in mouse models.
